# Mental and behavioral health characteristics among individuals injuriously shot by police in the United States

**DOI:** 10.1080/09638237.2025.2585191

**Published:** 2025-11-14

**Authors:** Julie A. Ward, Rebecca L. Fix, Javier A. Cepeda, Paul S. Nestadt, Cassandra K. Crifasi

**Affiliations:** a Department of Medicine, Health, and Society, Vanderbilt College of Arts and Science, Nashville, TN, USA; b Center for Research on Inequality and Health, Vanderbilt University, Nashville, TN, USA; c Department of Mental Health, Johns Hopkins Bloomberg School of Public Health, Baltimore, MD, USA; d Department of Epidemiology, Johns Hopkins Bloomberg School of Public Health, Baltimore, MD, USA; e School of Medicine, Baltimore, MD, USA; f Center for Gun Violence Solutions, Johns Hopkins Bloomberg School of Public Health, Baltimore, MD, USA; g Department of Health Policy and Management, Johns Hopkins Bloomberg School of Public Health, Baltimore, MD, USA

**Keywords:** Police violence, mental illness, public health, fatal and nonfatal shootings, substance use, use of force, crisis response

## Abstract

**Background:**

Criminalization of people experiencing mental illness is systemic, but the conditions surrounding police use-of-force in such encounters are under-examined.

**Aims:**

To describe mental or behavioral health (MBH) involvement in injurious shootings by U.S. police compared with MBH-uninvolved shootings.

**Methods:**

Using a 2015–2020 dataset developed from manual review of injurious shootings by police compiled from the Gun Violence Archive (GVA) (*n* = 10,615), we identified 2454 people shot in MBH-involved encounters. Through further review, we classified the MBH conditions and behaviors involved. Using descriptive statistics and logistic regression models, we compared characteristics of injured people, presenting conditions, and responses.

**Results:**

Twenty-three percent of injurious shootings by police involved MBH symptoms (*n* = 2336) or substance use (*n* = 921). Eighty-one percent of injured people threatened violence against others; 10% presented only self-harming symptoms, and 5% presented no symptoms. MBH involvement was associated with 1.5-times higher odds of fatality than MBH-uninvolved shootings and 31% higher odds of injuring an unarmed person *vs*. person with a gun. Clinician presence was identified in 1% of MBH-involved shootings.

**Conclusions:**

Police are de facto mental health system responders. Associated harms may be reduced through triage systems to facilitate clinician involvement, extreme risk protection order statutes, and better police training and protocols.

## Introduction

In 2022, an estimated 59.3 million U.S. adults, equating to 23% of the adult population, experienced a mental illness or substance use disorder (Substance Abuse and Mental Health Services Administration [SAMHSA], 2023). An estimated 6% of adults experienced serious mental illness with functional impairment (SAMHSA, 2023). Popular discourse frequently attributes extreme acts of violence, including mass shootings, to mental illness (Duxbury et al., 2018; McGinty, 2018; Metzl & MacLeish, 2015; Swartz & Bhattacharya, 2017). However, research indicates that, absent other risk factors (K. L. Johnson et al., 2016), violent or illegal behaviors among people experiencing mental illness are rare (Walsh & Fahy, 2002; Webermann & Brand, 2017). For example, data from 951 people recently discharged from a hospitalization for mental illness indicated these individuals were more likely to experience violent victimization (i.e. self-inflicted violence or violence by others) than to use violence against others. Reports of experiencing and using violence were more common among people who were unhoused, had prior arrests, or had co-occurring substance use (Monahan et al., 2017). This pattern of increased risk for violent victimization (versus perpetration) has also been observed among adults living with mental illness more generally (Desmarais et al., 2014).

Despite consistent patterns in victimization versus perpetration, criminal-legal system exposure among people experiencing mental illness remains a persistent problem. It has been exacerbated by the deinstitutionalized treatment of severe mental illness without sufficient replacement by community-based programs and an over-reliance on police and carceral institutions (Dvoskin et al., 2020; Egart, 2024; Gillooly & Thacher, 2024). The displacement of people with symptomatic mental or behavioral health (MBH) conditions from the healthcare system to the criminal legal system has multiple public health consequences. These include poorer treatment access; greater visibility of unmet social needs (Balfour & Zeller, 2023; Friedman, 2020; Hancock, 2024); and higher rates of police contact, arrests, and escalation to use-of-force among people with MBH conditions (Alang et al., 2021; Balfour & Zeller, 2023; Fuller et al., 2015; Laniyonu & Goff, 2021; National Alliance on Mental Illness, 2025; Parpouchi et al., 2021). Whether people with perceived MBH conditions experience more severe use of force, absent other factors such as weapon use or violent behaviors, is less clear (Chidgey et al., 2019; R. R. Johnson, 2011; Rossler & Terrill, 2017; Thomas et al., 2021). Limitations of related studies include reliance on data from select local agencies, owing to a lack of standardized use-of-force data collection nationally (Brault et al., 2024).

Few studies have examined use-of-force and MBH involvement on a national scale. In one analysis of fatal police shootings from 27 states participating in the National Violent Death Reporting System, an estimated 32% of encounters involved people with MBH symptoms (Khan et al., 2024). Ward et al. examined 6 years of national data, drawn from publicly available online sources and media reports, and estimated that 23% of all U.S. fatal and nonfatal shootings by police involved MBH symptoms. Sixty-seven percent of those shootings caused fatal injury, exceeding the 55% case fatality rate for all injurious shootings by police (Ward, Cepeda, Jackson, Johnson, et al., 2024). Still, relatively little is known about the circumstances of MBH-involved shootings and their precipitating encounters. Nationally representative descriptive analyses of such incidents may inform the development of more targeted prevention and response systems. The objective of this study was to describe the symptoms, situational characteristics, and responses associated with injurious shootings by police that involved co-occurring MBH indicators in the U.S. from 2015 to 2020, in comparison with shootings that did not.

## Methods

### Data

The data used for this analysis came from the Augmented Gun Violence Archive (a-GVA), a dataset that was constructed through manual review of shootings that were (1) listed as “officer-involved incidents” in the GVA that occurred between 1 January 2015 and 31 December 2020 and (2) determined to involve shots fired by law enforcement officer(s) that resulted in injury to a non-officer. The GVA is an online database of fatal and nonfatal shootings, identified daily from 7500 media, law enforcement, government, and other sources (Gun Violence Archive, 2025). The behavioral health module of the a-GVA was developed in two rounds. First, for each retained incident, a team of trained reviewers identified and reviewed publicly available materials, not limited to those linked to the GVA, to identify characteristics of the injured person, the precipitating incident, and the policing response. Reviewers were instructed to seek objective reflection of best available reporting, using any publicly available online sources. Details of the review process and quality assurance procedures are provided elsewhere (Ward, Cepeda, Jackson, Johnson, et al., 2024). Included among assessed characteristics was an item that asked reviewers to designate incidents that described the presence of MBH indicators (i.e. a mental health condition that was potentially relevant to the situation, dispatch related to mental illness, descriptions of suicidality, positive toxicology results, or other indications of intoxication).

In the second round of dataset development, MBH-designated incidents underwent further review to confirm MBH involvement and specify the mental health or substance use characteristics involved. In this round, reviewers examined publicly available, online materials including quotes by family members or acquaintances, toxicology results, and dispatch or encounter descriptions. This review was completed by five reviewers who received additional training and met weekly to ensure coding consistency. Assessed characteristics were abstracted using a Qualtrics instrument to produce a dataset that was ultimately merged onto the original a-GVA.

### Measures

Measures collected in the first round of dataset development, which are justified and described elsewhere (Ward, Cepeda, Jackson, Johnson, et al., 2024), included: shooting year and location; injury outcome; the injured person’s gender, socially assigned race and ethnicity, housing status, and weapon status at the time of the shooting; who initiated the encounter; the reason for the precipitating encounter; number of officers on-scene during the shooting; and type of agency whose officers fired shots. The geolocation and urbanicity of the nearest Zip Code Tabulation Area in the year of the shooting were also determined (Ward, Cepeda, Jackson, & Crifasi, 2024). Additionally, an indicator variable designating counties with no local newspapers in 2017, according to data compiled by the UNC Hussman School of Journalism and Media, was created (one or more local newspapers = 0, no local newspapers = 1) (UNC Hussman School of Journalism & Media, 2023). This variable was used to assess the significance of local news media presence on estimations of shootings by police with and without MBH involvement, a proxy for potential geographic disparities in under-specification of MBH indicators.

MBH items abstracted in the second-round review were determined *a priori* in consultation with experts in psychiatry and addiction medicine, crisis response systems, and police use of force. A codebook was developed for the team of non-clinician reviewers and iteratively refined for clarity and ease of use (Appendix A). Symptoms displayed by a person injured in an MBH-involved incident were classified by reviewers as: (1) suicidal or self-harming symptoms; (2) homicidal or violent symptoms; or (3) disorganized, paranoid, hallucinating, or bizarre behaviors. Multiple selections were possible. Separately, reviewers also indicated whether the incident was described as a completed or attempted “suicide by cop”. This determination was strictly based on police statements or explicit reporting, rather than reviewers’ subjective assessment of events.

For reported substance use, reviewers indicated the involvement of alcohol, methamphetamine or amphetamines, opioids, or other substances. These categories were mutually exclusive, but multiple selections were allowed. “Other” substances were described in a free-text field, which was subsequently reviewed and classified, as appropriate, by the first author. To assess the involvement of diagnosed conditions, reviewers indicated when an injured person was described as having an MBH diagnosis (e.g. schizophrenia, post-traumatic stress disorder, bipolar disorder, depression, unspecified diagnosis), a recent treatment lapse, or another condition that could have been perceived as an MBH indicator (e.g. hearing impairment, developmental disability, traumatic brain injury). An “other” free-text field was again provided and subsequently reviewed and classified by the first author. Multiple diagnostic categories for a single injured person were possible. Reviewers also indicated whether the person injured in an MBH-involved incident was described as having prior military experience.

Finally, measures describing MBH-involved incidents’ policing response included the location in which police fired shots (i.e. in a private residence, car, public or commercial setting, other, unknown), whether a clinician or other non-police co-responder was on-scene during the shooting, whether the shooting occurred following a prolonged standoff, and whether the precipitating incident (regardless of incident type) was described as a mental health crisis.

### Analysis

For the analysis, counts, proportions, and chi-square test statistics were calculated to compare incidents with and without MBH involvement (*N* = 10,615). Next, using unadjusted and adjusted logistic regression models with “unreported” or “unknown” variables excluded, we estimated odds of a given characteristic being associated with co-occurring MBH indicators when an injurious shooting by police was known to have occurred. All statistically significant variables from the univariate analyses were retained as covariates in the adjusted model. Confidence intervals were calculated based on an alpha of 0.05. Finally, to further describe the subset of MBH-involved incidents (*n* = 2454 injured people), counts and proportions were calculated for all second-round variables. For MBH symptoms, indicator variables were assigned to each of the three categories described above and used to create eight mutually exclusive new categories spanning all possible combinations of symptoms, including no symptoms. All analyses were performed using Stata version 16.1 (StataCorp, College Station, TX) (StataCorp, 2020). Ethical approval was obtained from the Johns Hopkins Bloomberg School of Public Health IRB (IRB00021322). Consent for participation did not apply, as the study was determined to be not human subjects research.

### Patient and public involvement

People with professional and personal caregiving expertise contributed to the design, conduct, analysis, and interpretation of data. People with lived experience were also included among the dataset development team of reviewers. The lead author met regularly with these contributors and encouraged sharing of thoughts and observations. All such contributions have been recognized with co-authorship or acknowledgement, as appropriate.

## Results

Of the 10,615 people injured in shootings by police in the U.S. from 2015 to 2020, co-occurring MBH indicators were identified in association with 2454 (23%). These shootings were evenly distributed across the study period, resulting in an average of 409 injured people each year. Sixty-seven percent of people (*n* = 1644) died from their injuries. In comparison, 52% of people injured in MBH-uninvolved shootings died from their injuries (*n* = 4230). Most injured people were cisgender men (94% of MBH-involved and MBH-uninvolved shootings). Forty-seven percent of people injured in an MBH-involved shooting (*n* = 1140) were identified as non-Hispanic White and 15% were non-Hispanic Black (*n* = 379). In comparison, 30% of people injured in MBH-uninvolved shootings were non-Hispanic White (*n* = 2468) and 23% were non-Hispanic Black (*n* = 1856). Race and ethnicity were unknown in 21% of MBH-involved shootings and 31% of shootings without MBH involvement (*n* = 524 and *n* = 2519, respectively). Nearly 5% of MBH-involved shootings (*n* = 121) resulted in injuries to people who were unhoused (*vs.* 2% of injurious shootings without MBH involvement, *n* = 157).

The Pacific region reported the largest portion of MBH-involved shootings (*n* = 550, 22%, followed by the South Atlantic region (*n* = 440, 18%). Forty-eight percent (*n* = 1171) of MBH-involved shootings occurred in Town or Rural Zip Codes (*vs*. 44% of MBH-uninvolved injurious shootings, *n* = 3558). A large majority of MBH-involved shootings followed events that were initiated by dispatch (MBH-involved: 82%, *n* = 2004; MBH-uninvolved: 55%, *n* = 4484). Police contact prior to an MBH-involved shooting most commonly originated as a response to a suicidal or behavioral health crisis (23%, *n* = 563). Domestic incidents (18%, *n* = 450); disorderly conduct, disputes, or disturbances (6%, *n* = 149), weapon complaints (5%, *n* = 133), wellbeing checks (4%, *n* = 91), and threats (3%, *n* = 74) were also notably represented among MBH-involved injurious shootings. Each of these incident types comprised a relatively larger portion of MBH-involved shootings by police than those without MBH involvement. At the time of the police shooting, slightly over half of injured people in MBH-involved incidents were armed with a gun or replica gun (gun: 49%, *n* = 1200; replica gun: 6%: *n* = 134), and 25% were armed with a knife (*n* = 618). This represents a lower prevalence of guns and a higher prevalence of knives compared with people injured in MBH-uninvolved shootings (gun: 56%, *n* = 4544; replica gun: 3%, *n* = 230; knife: 11%, *n* = 913) (Table 1).

After controlling for all significant variables, odds of fatality (*vs.* nonfatal injury) were 1.46 times higher in MBH-involved shootings compared to shootings without MBH involvement (95% CI: 1.27–1.68). Given that an injurious shooting by police occurred, when the incident was described as involving MBH indicators the adjusted odds of the injured person being non-Hispanic Black were 38% lower than non-Hispanic White (95% CI: 0.53–0.74); odds were 31% lower for Hispanic of any race (95% CI: 0.57–0.82). Compared with injured people who were housed, when an unhoused person was injured, odds of MBH involvement were 2.18-times higher (95% CI: 1.62–2.94).

Odds of an MBH-involved shooting being precipitated by a dispatch-initiated encounter were 1.66-times higher than an officer-initiated encounter (95% CI: 1.33–2.06); subject-initiated encounters were not statistically significantly associated with MBH-involvement. Compared with shots-fired precipitating events, incidents that were expected to be suicidal or behavioral health crises had the highest odds of ultimately being MBH-involved encounters (aOR: 35.38, 95% CI: 23.27–53.79). Other incident types that were also more likely to involve MBH concerns (vs. shots-fired precipitating incidents) were wellbeing checks (aOR: 5.11, 95% CI: 3.24–8.04), crashes (aOR: 2.35, 95% CI: 1.37–4.06), disorderly conduct or disturbances (aOR: 2.01, 95% CI: 1.45–2.79), weapon complaints (aOR: 1.71, 95% CI: 1.25–2.34), and threats (aOR: 1.66, 95% CI: 1.11-2.47). When someone was injured during an MBH-involved shooting by police, odds were 31% lower that the injured person was armed with a gun (*vs.* unarmed) as compared with people injured in MBH-uninvolved incidents (95% CI: 0.55–0.85). The odds of being armed with a blunt object were somewhat higher (aOR: 1.52, 95% CI: 1.01–2.28), and the odds of being armed with a vehicle were lower (aOR: 0.60, 95% CI: 0.42–0.84). After controlling for all other characteristics, odds of an MBH-involved shooting occurring in the South were somewhat lower than the referent region (New England). Year and county news desert designations were not statistically significantly associated with MBH involvement in injurious shootings by police (Table 2).

Table 3 describes the presenting and response-related characteristics of MBH-involved injurious shootings by police. In a large majority of these shootings (81%, *n* = 1983), injured people displayed violent symptoms directed toward others. Within this group, 34% of all injured people displayed other-directed violence only (*n* = 841), 29% displayed other-directed and self-harming behaviors (*n* = 705), 13% displayed other-directed violent symptoms and disorganized, paranoid, hallucinating, or bizarre behaviors (*n* = 319), and 5% displayed all three symptom types (*n* = 118). Ten percent of injured people displayed only suicidal or self-harming symptoms (*n* = 245); 4% displayed disorganized-type symptoms with or without self-harming (*n* = 108); 5% were not described as displaying any symptoms (*n* = 118). Separately, 16% of injured people (*n* = 382) were described as attempting “suicide by cop;” 64% of those shootings were fatal (data not shown).

Substance use was involved in 38% of MBH-involved injurious shootings (*n* = 921). The most common substance was alcohol (20%, *n* = 482), followed by methamphetamine (10%, *n* = 250). In 23% of MBH-involved shootings, the injured person was known to have an MBH diagnosis (*n* = 556), most of which were unspecified in reporting. A recent lapse in medication or other MBH management was described in five percent of incidents (*n* = 127). Half of MBH-involved shootings by police occurred in a private residence (*n* = 1233) and 39% occurred in a public or commercial setting (*n* = 959). A “mental health crisis” was identified in 34% of MBH-involved shootings (*n* = 821) and 32% of shooting encounters that were initiated by dispatch (*n* = 775, data not shown); approximately half of these incidents were dispatched as suicidal or behavioral health crises (*n* = 412, data not shown). Twenty-one percent of shootings involved a standoff situation (*n* = 507). A clinician or other non-police co-responder was on-scene for one percent of all MBH-involved injurious shootings by police (*n* = 21) (Table 3).

## Discussion

In this 6-year, nationwide analysis describing the extent and type of MBH involvement in fatal and nonfatal injurious shootings by police, we identified 2454 people injured in such shootings. These encounters comprised 23% of all injurious shootings by police, consistent with a prior estimate of mental illness involvement in all-cause fatal police encounters (Saleh et al., 2018). After controlling for other situational characteristics, people injured in shootings by police during MBH-involved incidents were 1.5-times more likely to die from their injuries than people injured in non-MBH encounters. Although over half of the people injured in these shootings were armed with a gun, victims of MBH-involved shootings were 31% more likely to be unarmed than victims of shootings without MBH involvement and were also more likely to be armed with a blunt object. However, other indications of violent threat could have been present. Our analysis suggests that 81% of MBH-involved injurious shootings by police described other-directed violence, such as charging at officers or verbally threatening to kill another person. Still, 19% of injured people were not described as posing a threat to others, including five percent with no reported MBH symptoms and ten percent who presented with self-harming behaviors alone. Prior studies suggest that stigmas against serious mental illnesses (e.g. those involving psychosis or delusions) or substance use may lead some officers to assume higher dangerousness or lower credibility (Dailey & Dubrow, 2024; Kruis et al., 2020; Stone et al., 2024; Watson et al., 2004). MBH-related stereotypes may also lead police to interpret aggression from non-compliance or feel lower levels of empathy for a person (Tartaro et al., 2021). Such expectations may not be improved by typical Crisis Intervention Training programs, despite observed gains in trained officers’ confidence, calling into question the adequacy of this common training intervention (Chidgey et al., 2019; Dailey & Dubrow, 2024; Tartaro et al., 2021).

Incidents that police initially anticipated to be suicidal or behavioral health crises accounted for 23% of all MBH-involved shootings nationally. Other such shootings were precipitated by a variety of incident types, suggesting that multiple indicators may be needed to accurately anticipate MBH involvement. Demographically, we found 47% of people injured in MBH-involved encounters were non-Hispanic White, 15% were non-Hispanic Black, and 13% were Hispanic of any race. For Black people, this distribution more closely resembles the demographics of the U.S. population overall as compared to MBH-uninvolved shootings or injurious shootings by police in general (US Census Bureau QuickFacts, n.d.; Ward, Cepeda, Jackson, & Crifasi, 2024; Ward, Cepeda, Jackson, Johnson, et al., 2024). This relatively lower representation of Black victims, atypical among studies of many policing-related harms (Alang et al., 2017; Esposito et al., 2021; GBD 2019 Police Violence US Subnational Collaborators, 2021; Jindal et al., 2022), may be a product of the Black-White mental health paradox–an observed pattern of lower rates of psychiatric disorders among Black people, particularly pronounced in age groups most represented among people injured in shootings by police (U.S. Census Bureau QuickFacts, 2024;; Ward, Cepeda, Jackson, & Crifasi, 2024). Alternatively, observed differences may reflect racialized disparities in media portrayals of police shooting victims, with White victims potentially receiving more sympathetic coverage that emphasizes mental health, as has been observed in portrayals of mass shooting perpetration (Duxbury et al., 2018). Either of these scenarios would suggest persistence of racial inequities, though they may be obscured.

In alignment with prior analyses of MBH-involved fatal encounters with police more broadly (Saleh et al., 2018), roughly half of the MBH-involved shooting injuries we analyzed occurred in a private residence, while 39% occurred in a public or commercial setting. Even after considering that many acts of violence do not lead to police involvement, and many police-involved acts of violence do not result in use of deadly force, this estimate suggests a possible overrepresentation of public encounters. In comparison, a large study of U.S. adults with mental illness found that when people with mental illness used violence, two-thirds (64%) of those acts occurred in residential settings and just 28% occurred in public contexts, including schools or workplaces (Desmarais et al., 2014). Prior research suggests that in publicly witnessed encounters, police may perceive a narrower range of decision-making options (J. D. Wood et al., 2017). Though speculative, absent other guidance, such perceived constraints may potentiate uses of force in public MBH responses. This may suggest an opportunity for more focused training and response protocols to guide safe and fair reactions to MBH concerns in public environments. Future research should further examine the trajectory of self-harming crisis calls, police encounters with unhoused people, and shootings described as “suicide by cop” to further inform injury prevention.

When we examined how MBH-involved policing encounters began, we found that all but 17% of MBH-involved shootings followed a non-officer-initiated encounter. Compared with shootings without MBH involvement, odds were 1.7-times higher the encounter was initiated by dispatch. Approximately one-third of MBH-involved dispatched encounters that led to injurious shootings were described as mental health crises, suggesting potential for dispatch-focused preventive interventions to be impactful. Yet only one percent of MBH-involved shootings described having a clinician on scene. While potentially reflective of the relative nascency of co-responder models nationally (Uding et al., 2025), this also suggests that dispatch services are but one important component of a larger crisis-response system. Moreover, MBH encounters with police are not limited to moments of crisis; situations that stem from more chronic vulnerabilities may be an under-appreciated function of police (J. D. Wood et al., 2017). For example, we found that a significantly larger portion of people injured in MBH-involved encounters – nearly 5% – were unhoused when they were shot during a policing encounter, evidence of acute-on-chronic MBH and social vulnerabilities. Anecdotally, alternative-responder programs may both directly (e.g. through street outreach) and indirectly (e.g., through department-based training and norms shifting) affect police involvement and approaches with people experiencing MBH symptoms, both in crisis and non-crisis scenarios (Origer & Norgaisse, 2023; Yang et al., 2025). Future research should examine whether the adoption of co-responder and other non-police crisis response models are associated with fewer injurious shootings by police.

### Limitations

To our knowledge, this study was the first to describe the specific involvement of MBH symptoms and conditions coinciding with fatal and nonfatal shootings by police based on a nationally inclusive dataset. Still, some limitations should be considered. First, although incident review was not restricted to GVA-linked media reports, journalistic sources were the primary contributor to a-GVA data. An overemphasis on police perspectives in reporting on police violence (Walker, 2022) may bias estimates of MBH involvement. Prior studies that used the a-GVA have demonstrated proportionate alignment of characteristics with other analyses of police shootings drawn from agency- or state-administrative data, offering assurance of overall data quality (Ward, Cepeda, Jackson, & Crifasi, 2024; Ward, Cepeda, Jackson, Johnson, et al., 2024). Still, our estimates may be conservative, and reporting depth and focus may differ by victim characteristics and incident location. The lack of a statistically significant association between local newspaper presence and the designation of MBH-involvement is an assuring indicator of balanced quality of reporting on this measure.

### Implications

Police play a de facto role in the U.S. public health system, with broad significance to people experiencing MBH conditions. This nationwide analysis of police-involved encounters that escalated to injurious shootings by police highlights multiple opportunities for harm reduction. Currently, 21 states, Washington DC, and the U.S. Virgin Islands have statutes allowing police or others to petition for the temporary removal of firearms when extreme risk of harm to self or others is indicated (Zeoli et al., 2025). Appropriate use of such a policy tool may reduce risk of harm from both the precipitating threat and from potentially heightened police perceptions of armed threat when co-occurring MBH symptoms are involved. Investing in evidence-informed dispatch systems and alternative responders (Gillooly, 2022; Ward et al., 2022; L. L. Wood et al., 2024) are additional mechanisms for reducing police encounters but are unlikely to be a panacea (Jones, 2024; Kirst et al., 2015). More effective training and procedural guidance for safer and fair police responses for people with MBH conditions are also needed (Soderstrom & Bumgarner, 2025). Future research should identify best prevention and treatment strategies for high-impact groups (Spolum et al., 2023) and evaluate such strategies’ effects on harm reduction and return on investment, to include nonfatal injuries.

## Supplementary Material

Supp 1

Supplemental data for this article can be accessed online at https://doi.org/10.1080/09638237.2025.2585191.

## Figures and Tables

**Table 1.** Characteristics of injurious shootings by police, 2015–2020, by mental or behavioral health (MBH) involvement (*N* = 10,615).

Characteristic	MBH involved (column %) *n* = 2454 (23.1)	MBH not involved (column %) *n* = 8161 (76.9)	Chi2
Year			

2015	394 (16.1)	1299 (15.9)	*p* = .283
2016	416 (17.0)	1285 (15.8)	
2017	399 (16.3)	1381 (16.9)	
2018	448 (18.3)	1399 (17.1)	
2019	401 (16.3)	1354 (16.6)	
2020	396 (16.1)	1443 (17.7)	

Shooting outcome			

Nonfatal	810 (33.0)	3931 (48.2)	*p* < .001
Fatal	1644 (67.0)	4230 (51.8)	

Injured person gender			

Cisgender man	2296 (93.6)	7686 (94.2)	*p* < .001
Cisgender woman	149 (6.1)	386 (4.7)	
Transgender	6 (0.2)	5 (0.1)	
Unknown	3 (0.1)	84 (1.0)	

Injured person race and ethnicity			

Non-Hispanic White	1140 (46.5)	2468 (30.2)	*p* < .001
Non-Hispanic Black	379 (15.4)	1856 (22.7)	
Asian or Pacific Islander	42 (1.7)	89 (1.1)	
Hispanic	318 (13.0)	1112 (13.6)	
American Indian or Alaska native	40 (1.6)	88 (1.1)	
Other or multiple	11 (0.5)	29 (0.4)	
Unknown	524 (21.4)	2519 (30.9)	

Housing status			

Housed or unstated	2333 (95.1)	8004 (98.1)	p <.001
Unhoused	121 (4.9)	157 (1.9)	

US census region^a^			

New England	77 (3.1)	161 (2.0)	*p* < .001
Mid-Atlantic	167 (6.8)	478 (5.9)	
East North Central	272 (11.1)	849 (10.4)	
West North Central	158 (6.4)	552 (6.8)	
South Atlantic	440 (17.9)	1662 (20.4)	
East South Central	150 (6.1)	697 (8.5)	
West South Central	324 (13.2)	1281 (15.7)	
Mountain	316 (12.9)	1053 (12.9)	
Pacific	550 (22.4)	1428 (17.5)	

Urbanicity^b^			

Urban ZIP Code	772 (31.5)	2822 (34.6)	*p* = .001
Suburban ZIP Code	511 (20.8)	1781 (21.8)	
Town or rural ZIP Code	1171 (47.7)	3558 (43.6)	

Response type			

Officer initiated	405 (16.5)	3486 (42.7)	*p* < .001
Dispatched	2004 (81.7)	4484 (54.9)	
Subject initiated	29 (1.2)	81 (1.0)	
Unreported	16 (0.7)	110 (1.4)	

Shooting agency			

Local police	1540 (62.8)	4996 (61.2)	*p* < .001
Sheriff’s office	598 (24.4)	1851 (22.7)	
State police	120 (4.9)	462 (5.7)	
Special jurisdiction	12 (0.5)	69 (0.9)	
Constable or marshal	3 (0.1)	8 (0.1)	
Federal agency	11 (0.5)	172 (2.1)	
Multiple agency types	153 (6.2)	533 (6.5)	
Unknown	17 (0.7)	70 (0.9)	

Number of officers on scene at time of shooting			

One	329 (13.4)	1353 (16.6)	*p* < .001
Multiple	2089 (85.1)	6547 (80.2)	
Unknown	36 (1.5)	261 (3.2)	

Weapon status of the injured person			

Gun or replica gun	1346 (54.8)	4792 (58.7)	*p* < .001
Gun	1200 (48.9)	4544 (55.7)	
Replica gun	134 (5.5)	230 (2.8)	
Knife	618 (25.2)	913 (11.2)	
Blunt object	75 (3.1)	124 (1.5)	
Vehicle	83 (3.4)	691 (8.5)	
Other, including multiple without a gun	69 (2.8)	170 (2.1)	
Unarmed	203 (8.3)	752 (9.2)	
Unknown	60 (2.4)	719 (8.8)	

Precipitating incident type			

Suicidal or behavioral health crisis	563 (22.9)	68 (0.8)	*p* < .001
Assault	102 (4.2)	309 (3.8)	
Burglary	35 (1.4)	193 (2.4)	
Carjacking or robbery	74 (3.0)	774 (9.5)	
Disorderly conduct, dispute, or disturbance	149 (6.1)	260 (3.2)	
Domestic disturbance, dispute, or violence	450 (18.3)	1178 (14.4)	
Investigative	48 (2.0)	530 (6.5)	
Shooting	175 (7.1)	818 (10.0)	
Stolen vehicle	10 (0.4)	138 (1.7)	
Suspicious person or vehicle	106 (4.3)	457 (5.6)	
Threats	74 (3.0)	142 (1.7)	
Traffic stop	199 (8.1)	1474 (18.1)	
Trespassing	41 (1.7)	141 (1.7)	
Warrant or arrest	82 (3.3)	920 (11.3)	
Weapon complaint	133 (5.4)	322 (4.0)	
Wellbeing check	91 (3.7)	65 (0.8)	
Other^c^	102 (4.2)	257 (3.2)	
Unreported or unknown	20 (0.8)	115 (1.4)	

County news desert (2017)			

No local newspapers	52 (2.1)	213 (2.6)	p = .172

a Regions defined according to US Census Bureau. New England includes CT, ME, MA, NH, RI and VT; Mid-Atlantic includes NH, NY, and PA; East North Central includes IN, IL, MI, OH, WI; West North Central includes IA, KS, MN, MO, NE, ND, and SD; South Atlantic includes DE, DC, FL, GA, MD, NC, SC, VA, and WV; East South Central includes AL, KY, MS, and TN; West South Central includes AR, LA, OK, and TX; Mountain includes AZ, CO, ID, NM, MT, UT, NV, and WY; Pacific includes AK, CA, HI, OR, and WA.

b ZIP Code Tabulation Area designation defined by the National Center for Education Statistics in the year of the shooting. Aggregation methods detailed elsewhere. (Ward, Cepeda, Jackson, & Crifasi, 2024)

c Incidents described as “other” include vandalism, arson, pedestrian stop, hostage, and others.

**Table 2.** Odds of mental or behavioral health involvement among injurious shootings by police, by incident and person characteristics.

Mental or behavioral health incident characteristics	Unadjusted odds ratio (OR)	95% CI	Adjusted odds ratio (aOR)	95% CI
Year				

2015	*Ref*		–	–
2016	1.07	0.91, 1.25	–	–
2017	0.95	0.81, 1.12	–	–
2018	1.06	0.90, 1.23	–	–
2019	0.98	0.83, 1.14	–	–
2020	0.90	0.77, 1.06	–	–

Shooting outcome				

Nonfatal	*Ref*		*Ref*	
Fatal	1.89***	1.72, 2.07	1.46***	1.27, 1.68

Injured person gender				

Cisgender man	*Ref*		*Ref*	
Cisgender woman	1.29**	1.06, 1.57	1.25	0.94, 1.65
Transgender	4.02*	1.22, 13.17	1.72	0.37, 8.11

Injured person race and ethnicity				

Non-Hispanic White	*Ref*		*Ref*	
Non-Hispanic Black	0.44***	0.39, 0.50	0.62***	0.53, 0.74
Asian or Pacific Islander	1.02	0.70, 1.48	0.84	0.54, 1.30
Hispanic	0.62***	0.54, 0.71	0.69***	0.57, 0.82
American Indian or Alaska Native	0.98	0.67, 1.44	0.97	0.62, 1.53
Other or multiple	0.82	0.41, 1.65	0.88	0.41, 1.88

US Census regiona				

New England	*Ref*		*Ref*	
Mid-Atlantic	0.73	0.53, 1.01	0.78	0.50, 1.22
East North Central	0.67**	0.49, 0.91	0.81	0.54, 1.23
West North Central	0.60**	0.43, 0.83	0.78	0.50, 1.21
South Atlantic	0.55***	0.41, 0.74	0.67*	0.45, 0.99
East South Central	0.45***	0.33, 0.62	0.61*	0.39, 0.94
West South Central	0.53***	0.39, 0.71	0.60*	0.40, 0.90
Mountain	0.63**	0.47, 0.85	0.76	0.51, 1.14
Pacific	0.81	0.60, 1.07	0.96	0.64, 1.42

Urbanicity^b^				

Urban ZIP code	*Ref*		*Ref*	
Suburban ZIP code	1.05	0.92, 1.19	1.00	0.84, 1.20
Town or rural ZIP code	1.20***	1.08, 1.33	1.10	0.94, 1.29

Housing status				

Housed or unstated	*Ref*		*Ref*	
Unhoused	2.64***	2.08, 3.37	2.18***	1.62, 2.94

Shooting agency				

Local police	*Ref*		*Ref*	
Sheriff’s office	1.05	0.94, 1.17	0.89	0.75, 1.05
State police	0.84	0.68, 1.04	1.00	0.75, 1.34
Special jurisdiction	0.56	0.30, 1.04	1.06	0.46, 2.47
Constable or marshal	1.22	0.32, 4.59	1.30	0.23, 7.24
Federal agency	0.21***	0.11, 0.38	0.65	0.33, 1.29
Multiple agency types	0.93	0.77, 1.12	1.08	0.84, 1.39

Response type				

Officer initiated	*Ref*		*Ref*	
Dispatched	3.85***	3.43, 4.32	1.66***	1.33, 2.06
Subject initiated	3.08***	1.99, 4.77	1.64	0.93, 2.90

Number of officers on scene at time of shooting				

One	*Ref*		*Ref*	
Multiple	1.31***	1.15, 1.49	1.18	0.99, 1.41

Weapon status of the injured person				

Unarmed	*Ref*		*Ref*	
Gun or replica gun	1.04	0.88, 1.23	0.69***	0.55, 0.85
Knife	2.51***	2.08, 3.02	1.27	0.99, 1.63
Blunt object	2.24***	1.62, 3.10	1.52*	1.01, 2.28
Vehicle	0.44***	0.34, 0.59	0.60**	0.42, 0.84
Other, including multiple without a gun	1.50*	1.09, 2.07	1.30	0.88, 1.92

Precipitating incident type				

Shooting	*Ref*		*Ref*	
Assault	1.54**	1.17, 2.04	1.02	0.73, 1.43
Burglary	0.85	0.57, 1.26	0.52*	0.32, 0.86
Carjacking or robbery	0.45***	0.33, 0.60	0.49***	0.35, 0.68
Crash, including hit-and-run	2.17***	1.39, 3.39	2.35**	1.37, 4.06
Disorderly conduct, dispute, or disturbance	2.68***	2.07, 3.47	2.01***	1.45, 2.79
Domestic disturbance, dispute, or violence	1.79***	1.47, 2.17	1.29*	1.01, 1.64
Investigative	0.42***	0.30, 0.59	0.54**	0.35, 0.82
Stolen vehicle	0.34***	0.17, 0.66	0.53	0.24, 1.14
Suicidal or behavioral health crisis	38.70***	28.67, 52.24	35.38***	23.27, 53.79
Suspicious person or vehicle	1.08	0.83, 1.42	1.02	0.73, 1.43
Threats	2.44***	1.76, 3.37	1.66*	1.11, 2.47
Traffic stop	0.63***	0.51, 0.79	0.93	0.68, 1.28
Trespassing	1.36	0.93, 2.00	0.88	0.54, 1.42
Warrant or arrest	0.42***	0.32, 0.55	0.56**	0.39, 0.81
Weapon complaint	1.93***	1.49, 2.50	1.71***	1.25, 2.34
Wellbeing check	6.54***	4.58, 9.36	5.11***	3.24, 8.04
Other^c^	1.73***	1.26, 2.39	1.23	0.81, 1.85

County news desert				

Newspaper presence	*Ref*		–	–
No local newspapers	0.81	0.59, 1.10	–	–

*** *p* ≤ .001.

** *p* ≤ .01.

* *p* ≤ .05.

– indicates non-inclusion in adjusted models, owing to statistical non-significance in univariate models.

a Regions defined according to US Census Bureau. New England includes CT, ME, MA, NH, RI, and VT; Mid-Atlantic includes NH, NY, and PA; East North Central includes IN, IL, MI, OH, WI; West North Central includes IA, KS, MN, MO, NE, ND, and SD; South Atlantic includes DE, DC, FL, GA, MD, NC, SC, VA, and WV; East South Central includes AL, KY, MS, and TN; West South Central includes AR, LA, OK, and TX; Mountain includes AZ, CO, ID, NM, MT, UT, NV, and WY; Pacific includes AK, CA, HI, OR, and WA.

b ZIP code tabulation area designation defined by the National Center for Education Statistics in the year of the shooting. Aggregation methods detailed elsewhere. (Ward, Cepeda, Jackson, & Crifasi, 2024).

c Incidents described as “other” include vandalism, arson, pedestrian stop, hostage, and others.

**Table 3.** Characteristics of mental or behavioral health involved injurious shootings by police, 2015–2020 (*n* = 2454).

Characteristic	Injured people (column %) *n* = 2454
Mental or behavioral health symptoms involved	

No symptoms described	118 (4.8)
Suicidal or self-harming only	245 (10.0)
Homicidal or violent only	841 (34.3)
Disorganized, paranoid, hallucinating or bizarre behaviors only	81 (3.3)
Suicidal or self-harming AND homicidal or violent	705 (28.7)
Suicidal or self-harming AND disorganized, paranoid, hallucinating, or bizarre behaviors	27 (1.1)
Homicidal or violent AND disorganized, paranoid, hallucinating, or bizarre behaviors	319 (13.0)
Suicidal or self-harming AND homicidal or violent AND disorganized, paranoid, hallucinating, or bizarre behaviors	118 (4.8)

Incident was described as “Suicide by Cop” attempt	

Yes	382 (15.6)

Substance use involved	

Any substance	921 (37.5)
Alcohol use	482 (19.6)
Methamphetamine use	250 (10.2)
Opioid use	83 (3.4)
Cocaine use	44 (1.8)
Other^a^	144 (5.9)
Unspecified	77 (3.1)

Diagnosed conditions	

Any MBH diagnosis	556 (22.7)
Schizophrenia or schizoaffective disorder	160 (6.5)
Post-traumatic stress disorder	85 (3.5)
Bipolar disorder	105 (4.3)
Depression	132 (5.4)
Anxiety	24 (1.0)
Substance use disorder	77 (3.1)
Unspecified diagnosis	383 (15.6)
Lapse in medication or other management	127 (5.2)
Other symptomatic diagnosis (not MBH)	104 (4.2)

Military service history described for injured person	

No or unknown	2335 (95.2)
Yes	119 (4.9)

Mental health crisis	

No or unknown	1633 (66.5)
Yes	821 (33.5)

Clinician responder onsite	

No or unknown	2433 (99.1)
Yes	21 (0.9)

Location of shooting	

Private residence	1233 (50.2)
In car	228 (9.3)
In public or commercial setting	959 (39.1)
Other	24 (1.0)
Unknown	10 (0.4)

Standoff situation	

No or unknown	1949 (79.3)
Yes	507 (20.7)

Shooting occurred at or during transport to treatment facility	

No	2392 (97.5)
Yes	62 (2.5)

a Other substances include hallucinogens (e.g. PCP, LSD), sedatives (e.g. Benzodiazepines), Cannabinoids, Ecstasy, Ketamine, and others.
