# Supplementary material for: Mental and behavioral health characteristics among individuals injuriously shot by police in the United States
Source: J Ment Health. Author manuscript; Available in PMC 2025 Dec 23. (PMC12721205; doi:10.1080/09638237.2025.2585191)
Supplement: Supp 1 [file NIHMS2123800-supplement-Supp_1.pdf]

Mental and behavioral health characteristics among individuals injuriously shot by police in the United States

Julie A. Ward, Rebecca L. Fix, Javier A. Cepeda, Paul S. Nestadt, and Cassandra K. Crifasi

Journal of Mental Health

Appendix A. Codebook of Mental and Behavioral Health Characteristics

**Round 1 Variable**	**Definition and Guidance**
**Did the incident involve mental or behavioral health needs?**	A yes indicates any of the following were described:<br>• Mental health condition potentially relevant to the situation,<br>• Dispatch related to mental health, including wellness check, suicidal threat, suicidal ideation, etc.<br>• Objective toxicology results potentially relevant to the situation<br>• Reported substance use*<br>• Suicidal or self-harming symptoms<br>• "Suicide-by-cop" attempt<br>*Note: Substance involvement that did not involve intoxication (e.g., drug investigation or drug sales) does not qualify.
**Round 2 Variable**	**Definition and Guidance**
**Alcohol use**	Use described or indicated by >0.08 BAC
**Opioid use**	Substances include: heroin, fentanyl, morphine, methadone, many prescription pain medications.<br>Use described, indicated by positive result on reported toxicology results, or implied by report of Naloxone successfully reversing an overdose.
**Methamphetamine or amphetamine use**	Use described or indicated by positive result on reported toxicology results
**Other substance use (list)**	This free text field was subsequently reviewed and categorized by RN co-author
**Suicidal or self-harming symptoms**	A yes indicates the person was described as showing such symptoms.
**Homicidal or violent symptoms**	A yes indicates the person was described as showing such symptoms.

**Disorganized, paranoid, hallucinating, or bizarre behavioral symptoms**	A yes indicates the person was described as showing such symptoms.
**Mental illness diagnosis**	Free text field provided to indicate reported diagnoses (e.g., depression, schizophrenia, bipolar disorder, PTSD).
**Other symptomatic diagnosis**	Free text field provided to indicate symptoms of physical or developmental disability described (e.g., sensory impairment, dementia, acute symptoms of diabetes or seizure disorder, autism spectrum)
**"Suicide by cop" attempt**	A yes indicates that the incident was described as such, typically in a police statement or as reported by a journalist. Includes descriptions described as "suspected." Reviewers were instructed to indicate yes based on documented statements, not subjective review.
**Lapse in medication or other management**	A yes indicates that a family member or other acquaintance described the injured person as recently changing behaviors that were previously successful for managing a mental or behavioral health condition (e.g., stopped taking a medication, participating in therapy).
**Transport-related facility call**	A yes indicates the incident occurred during actual or attempted transportation assistance.
**Non-transport-related facility call**	A yes indicates the incident occurred at a hospital, outpatient treatment facility, assisted living facility, etc.
**Standoff situation**	A yes indicates the shooting occurred after a prolonged stand-off with police (e.g., police were called to a crime-related situation that culminated in an attempted "suicide by cop.")
**"Mental health crisis"**	Were officers called to a "mental health crisis," either by dispatch or by a bystander? A yes indicates these or similar words were used to describe why police came to the scene.
**Veteran involved**	A yes indicates one or more of the injured people was described as a veteran or person with other history of military service.
**Location of person injured in the shooting**	Reviewers selected whether the shooting occurred when the injured person was:<br>a) In a private residence, including a yard or standing in a driveway<br>b) In a vehicle<br>c) In a public or commercial space (e.g., hotel, hospital, assisted living facility)<br>d) In another location not listed in a, b, or c (including multiple different locations)

	e) In an unknown location (i.e., the physical context of the shooting was not described)
**Was a social worker, behavioral health clinician, or "co-responder" present with police?**	A yes indicates a social worker, behavioral health responder, or other "co-responder" or clinician was present with police. A free text field was provided to specify the role of the non-police responder(s) present at the time of the shooting.
